# Zebrafish: Housing and husbandry recommendations

**DOI:** 10.1177/0023677219869037

**Published:** 2019-09-11

**Authors:** Peter Aleström, Livia D’Angelo, Paul J Midtlyng, Daniel F Schorderet, Stefan Schulte-Merker, Frederic Sohm, Susan Warner

**Affiliations:** 1 Faculty of Veterinary Medicine, Norwegian University of Life Sciences, Oslo, Norway; 2 Department of Veterinary Medicine and Animal Productions, University of Naples Federico II, Naples, Italy; Italian Association of Laboratory Animal Sciences (AISAL); Stazione Zoologica Anton Dohrn, Naples, Italy; 3 Institute for Research in Ophthalmology, University of Lausanne and Ecole Polytechnique Fédérale of Lausanne, Sion, Switzerland; 4 Institute for Cardiovascular Organogenesis and Regeneration, WWU Münster, Faculty of Medicine, Münster, Germany; 5 CiM Cluster of Excellence, Faculty of Medicine, Münster, WWU Münster, Münster, Germany; 6 UMS AMAGEN, CNRS, INRA, Université Paris-Saclay, Gif sur Yvette, France; 7 Karolinska Institutet, Comparative Medicine, Stockholm, Sweden

**Keywords:** guidelines, housing, husbandry, laboratory animal welfare, zebrafish

## Abstract

This article provides recommendations for the care of laboratory zebrafish (*Danio rerio*) as part of the further implementation of Annex A to the European Convention on the protection of vertebrate animals used for experimental and other scientific purposes, EU Commission Recommendation 2007/526/EC and the fulfilment of Article 33 of EU Directive 2010/63, both concerning the housing and care of experimental animals. The recommendations provide guidance on best practices and ranges of husbandry parameters within which zebrafish welfare, as well as reproducibility of experimental procedures, are assured.

Husbandry procedures found today in zebrafish facilities are numerous. While the vast majority of these practices are perfectly acceptable in terms of zebrafish physiology and welfare, the reproducibility of experimental results could be improved by further standardisation of husbandry procedures and exchange of husbandry information between laboratories. Standardisation protocols providing ranges of husbandry parameters are likely to be more successful and appropriate than the implementation of a set of fixed guidance values neglecting the empirically successful daily routines of many facilities and will better reflect the wide range of environmental parameters that characterise the natural habitats occupied by zebrafish.

A joint working group on zebrafish housing and husbandry recommendations, with members of the European Society for Fish Models in Biology and Medicine (EUFishBioMed) and of the Federation of European Laboratory Animal Science Associations (FELASA) has been given a mandate to provide guidelines based on a FELASA list of parameters, ‘Terms of Reference’.

## Résumé

Cet article fournit des conseils sur les soins à apporter aux poissons-zèbres (*Danio rerio*) de laboratoire dans le cadre de la mise en œuvre de l’Annexe A de la Convention européenne sur la protection des animaux vertébrés utilisés à des fins expérimentales ou à d'autres fins scientifiques, de la Recommandation de la Commission Européenne 2007/526/CE et du respect de l’Article 33 de la Directive européenne 2010/63, concernant l’hébergement et les soins prodigués aux animaux d'expérimentation. Ces recommandations fournissent des orientations sur les meilleures pratiques et les plages de paramètres d’élevage à utiliser dans le cadre du bien-être des poissons-zèbres, afin d'assurer la reproductibilité des procédures expérimentales. Les procédures d’élevage qui existent de nos jours dans les installations utilisant des poissons-zèbres sont variées. Bien que la grande majorité de ces pratiques soit tout à fait acceptable pour la physiologie et le bien-être des poissons-zèbres, la reproductibilité des résultats d'expérience pourrait être améliorée si les procédures d’élevage étaient harmonisées et si les laboratoires échangeaient des informations concernant l’élevage. Dans le but d'harmoniser les pratiques d'élevages, des plages de paramètres acceptables ont été définies qui tiennent compte des pratiques actuelles et du vaste éventail de paramètres qui caractérise les habitats naturels occupés par les poissons-zèbres. De telles plages nous semblent plus appropriées que des valeurs fixes et sont plus faciles à adopter par les animaleries sans bouleverser l'ensemble de leur pratique quotidienne. Un groupe de travail conjoint, constitué de membres de l'European Society for Fish Models in Biology and Medicine (EUFishBioMed) et de la Federation of European Laboratory Animal Science Associations (FELASA), a été mandaté pour fournir des recommandations en matière d'hébergement et d'élevage des poissons-zèbres suivant la liste des paramètres de référence de la FELASA.

## Zusammenfassung

Dieser Artikel enthält Empfehlungen für die Pflege von Zebrafischen (*Danio rerio*) im Forschungslabor im Rahmen der weiteren Umsetzung des Anhangs A des Europäischen Übereinkommens zum Schutz der für Versuche und andere wissenschaftliche Zwecke verwendeten Wirbeltiere, der Empfehlung 2007/526/EG der EU-Kommission und der Erfüllung von Artikel 33 der EU-Richtlinie 2010/63, sowohl für die Haltung als auch Pflege von Versuchstieren. Die Empfehlungen enthalten Leitlinien basierend auf bewährten Verfahren und definieren Haltungsparameter, in denen das Wohlergehen der Zebrafische sowie die Reproduzierbarkeit der experimentellen Verfahren gewährleistet sind. Momentan werden in Zebrafischanlagen viele verschiedene Haltungsverfahren genutzt. Während die überwiegende Mehrheit dieser Verfahren in Bezug auf die Physiologie und das Wohlergehen der Zebrafische insgesamt akzeptabel ist, könnte die Reproduzierbarkeit der experimentellen Ergebnisse durch eine weitere Standardisierung der Haltungsabläufe und den Austausch von Informationen zwischen den Laboren verbessert werden. Standardisierungsprotokolle, die eine Bandbreite von Haltungsparametern vorsehen, dürften erfolgversprechender und sachdienlicher sein als die Umsetzung einer Reihe fixer Richtwerte, die empirisch bewährte Abläufe in vielen Einrichtungen vernachlässigen würden, und die Vielfalt der Umweltkenngrößen, die die natürlichen Lebensräume von Zebrafischen charakterisieren, nicht ausreichend widerspiegeln. Mitglieder der European Society for Fish Models in Biology and Medicine (EUFishBioMed) und der Federation of European Laboratory Animal Science Associations (FELASA) wurden beauftragt, Leitlinien auf der Grundlage einer FELASA-Parameterliste „Terms of Reference“ zu erstellen.

## Resumen

Este artículo da recomendaciones sobre el cuidado del pez cebra (*Danio rerio*) de laboratorio como parte de una mayor implementación del Anexo A a la Convención Europea sobre la protección de animales vertebrados usados para fines experimentales y científicos, Recomendación de la Comisión Europea 2007/526/CE, y el Artículo 33 de la Directiva de la UE 2010/63, ambas sobre el enjaulamiento y el cuidado de animales de experimentación. Las recomendaciones ofrecen una guía sobre las mejores prácticas y los tipos de parámetros de cría respecto al bienestar del pez cebra, y garantizan la reproducibilidad de los procedimientos experimentales. Los procedimientos de cría actuales en las instalaciones de peces cebra son numerosos. A pesar de que la gran mayoría de estas prácticas son perfectamente aceptables en cuanto a la fisiología y bienestar del pez cebra, la reproducibilidad de los resultados experimentales podría mejorarse mediante una mayor estandarización de los procedimientos de cría y el intercambio de información pertinente entre laboratorios. Los protocolos de estandarización que ofrecen rangos de parámetros para la cría y la estabulación serán más adecuados que la implementación de una serie de valores fijos que no incluyen los parámetros satisfactórios usados rutinariamente en muchas instalaciones. Además, los protocolos de estandarización reflejarán mejor la amplia gama de parámetros medioambientales que caracterizan los hábitats naturales ocupados por el pez cebra. Un grupo de trabajo en conjunto que estudia las recomendaciones sobre jaulas y cría del pez cebra, junto con miembros de la Sociedad Europea para los Modelos de Peces usados en Biología y Medicina (EUFishBioMed) y la Federación Europea de Asociaciones Científicas de Animales de Laboratorio (FELASA), ha ofrecido directrices en base a la lista de parámetros de FELASA, «Términos de referencia».

## Background

### The zebrafish model

Zebrafish (*Danio rerio*) are increasingly used to address key questions raised in basic and applied research including, but not limited to, biomedicine, toxicology, environmental science, biotechnology and aquaculture.^1^ Zebrafish have proven well-suited to large-scale genetic screening, whether in search of models for human disease or for control mechanisms related to natural or pathological differentiation of cells and tissues, studies which are more difficult to carry out in mammalian model organisms because of their long *in-utero* gestation periods.^2^

A long list of established zebrafish models aids in understanding the mechanisms behind diseases, and novel toxicology models have been developed for endocrine disruptor studies.^3,4^ Many clinically relevant biochemical pathways are conserved between fish and human, but cannot be reliably investigated *in vitro*, in unicellular organisms, or in invertebrates.

Worldwide, more than 1000 laboratories use zebrafish as a research model (*zfin.org*). In Europe, the tightly interconnected zebrafish scientific community consists of more than 350 laboratories (*eufishbiomed.eu*) that would benefit from guidelines for standardised husbandry conditions at the European/international level.^5,6^ The guidelines provided here are meant to constitute a framework for European zebrafish research groups, and a basis and reference point for future welfare relevant studies.

### The biology of zebrafish

**Figure 1.**
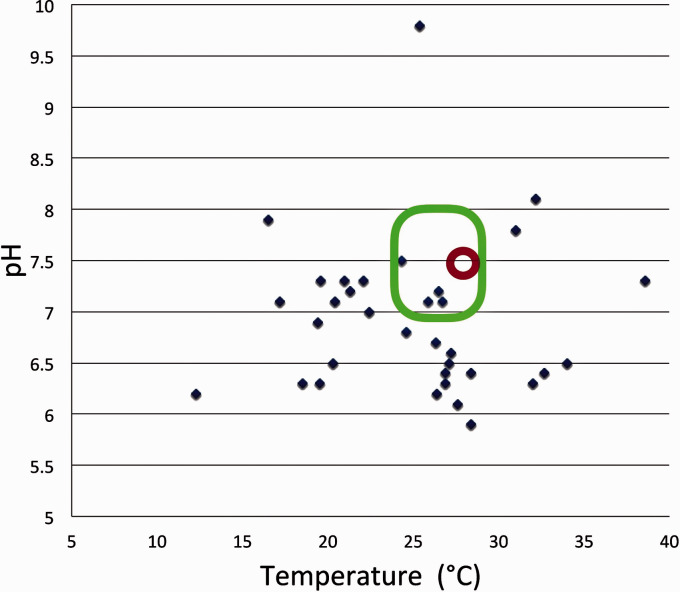
Temperatures and pH levels measured at 35 natural zebrafish habitats at altitudes between 14 m and 1576 m above sea level (blue dots).^7,8^ Ranges recommended for zebrafish housing systems (pH 6.5–8 and 24–29℃; green area) and values commonly referred to in literature being optimal for reproduction (pH 7.4–7.5 and 28℃; red circle) are indicated.

Zebrafish are native to South Asia where they are found in streams, ponds and other slow-flowing water bodies including rice fields. The range of environmental conditions under which zebrafish live includes water bodies with temperatures ranging from below 10℃ up to 40℃, clear or turbid water, pH from 6 to just below 10, conductivities from 10 to 271 µS/cm, depth from surface level down to 60 cm, a wide range of bottom substrates (primarily silt), presence or absence of vegetation and elevations ranging from sea level to over 1500 m altitude (Figure 1).^7,8^ Predation pressure led to the development of shoaling behaviour,^9,10^ believed to reduce stress and aggression among fish held in small groups. Of course, while conditions in the wild can vary considerably, attempts have been made to provide stable husbandry conditions in laboratory settings. It is important to emphasise that when implementing lessons from fish in their natural habitats, some caution is warranted: laboratory strains have been maintained in research facility aquaria for at least 25 years (*c.* 100 generations). During this time, diverse adaptations and domestication steps^10^ have arisen, for example concerning mechanisms of sex determination.^11^ Historically, combined knowledge from research on zebrafish in the wild together with experiences acquired from keeping and breeding them in captivity has set the foundation for today’s zebrafish husbandry. Several laboratory manuals that provide recommendations for zebrafish care and good husbandry practices are available.^12–14^ In addition, a vast quantity of internet-based resources is available to aid both beginners and more experienced researchers using the zebrafish model (*zfin.org*).^15^

### The Directive 2010/63/EU

The 3Rs (Refinement, Reduction and Replacement) are an intrinsic part of the EU Directive 2010/63 on the protection of animals used for scientific purposes and apply to all non-human vertebrate animals, including independently feeding larval forms (Article 1). However, fish are not elaborated on and specifics for zebrafish are not provided at all. This motivates the work on common European guidelines for ensuring sound husbandry and high standards of welfare for zebrafish.

A key issue within the Directive is the time point when fish larvae can be regarded as independently feeding and free-living, thus falling under the scope of the legislation. The development of poikilothermic animals is temperature dependent, and for zebrafish this critical time point is 120 hours post fertilisation (hpf) at 28.5℃.^16,17^

### International exchange of experience and best practices

During the last few years, dedicated husbandry sessions have become mandatory at international zebrafish conferences.^6^ Two recent international surveys on zebrafish husbandry practices from over 100 facilities in five continents showed that parameters like physical housing, nutrition, pathogen status, water conditions and holding densities can be highly variable.^18,19^ In addition, international zebrafish resource centres and several single facilities have recently published husbandry procedures that lay the groundwork for educated discussions for a gradual standardisation of zebrafish protocols.^20–25^ Although less common in earlier literature, there is now an increasing trend towards including detailed information about the husbandry parameters used in studies in the Materials and Methods section of publications. With the notion that parameters like feeding, temperature, dark–light period etc. may affect results from experimental research,^26^ it is recommended to always include a detailed description of the zebrafish husbandry parameters used. Important factors for dissemination of experiences and skills are national/regional/international training initiatives with theoretical and hands-on courses.^27^

## Recommendations for housing and husbandry

The guidelines provided here address and suggest recommendations for: transportation and reception, safe introduction into facilities, housing systems and environmental conditions (temperature, dark–light cycle, water quality, stocking density, environmental enrichment, feeding), breeding, identification and nomenclature as well as sanitisation of equipment. An overview of our recommendations together with relevant literature references for housing and husbandry are summarised in Table 1. For some aspects, the current scientific literature either lacks consensus, or parameters have been tested on a small-scale level only and for a limited period of time, without, for example, evaluating effects on the next generation. We decided in those instances not to provide a recommendation and rather emphasise the need for further work. We trust these guidelines will not only be useful for zebrafish users/scientists, but also serve as a reference and guideline for authorities and veterinarians serving the aquarist communities.

**Table 1.** Summary of recommendations for housing and care of zebrafish colonies (EU Directive 2010/63 Article 33 and Annex III). Conditions outside the proposed ranges may be acceptable for time-limited studies, trials and experiments carried out following evaluation and authorisation of projects (EU Directive 2010/63, section III).

Issue	Recommendations	Refs
General	‘Materials and Methods’ sections in publications should include sufficient detail of general husbandry parameters, including a description of the health monitoring, water chemistry, housing systems, fish stocking density, water temperature, dark–light cycle, fish feed and feeding frequency. Apply experimental design with power analysis to define the minimum number of data/fish needed to allow statistically significant results.	
Transportation and reception	Whenever possible, relevant documentation from the exporting facility should be sent prior to the shipment. Plan for the shortest possible shipment time. Only bleached embryos and/or healthy juveniles/adult fish can be shipped. Upon arrival, visually check the animals, equilibrate watertemperature and transfer the fish into the reception tank without transferring the water from the transport container. In order to avoid ammonia poisoning, it is vital that adult fish are removed with a net from the transport container immediately after temperature adaptation.	12, 14, 25, 28
Safe Introduction into facilities – Quarantine	Use a quarantine system for raising and monitoring newly imported embryos and adult fish, and ask for health certificate from the sending facility. Introduce only disinfected/bleached embryos from healthy parents into the main facility. Never introduce adult fish from an external provider into the main facility.	14,
		28



Housing systems	All recirculating water systems for zebrafish should include filter systems, water chemistry monitoring or regulation capabilities, germicidal UV irradiation, light and temperature control units.	25, 29
Temperature	For standard growth curves, embryos should be kept at a temperature of 28.5 ± 0.5℃ to 120 hpf. Adjusting the rearing and maintenance temperatures for larvae and adults within a 24–29℃ range is recommended. For time-limited experiments a temperature range from 15 to 39℃ is acceptable.	29, 30,
		32, 33



Dark–light cycle	For larvae and adults, use a standardised static dark–light cycle.	35, 36
Water quality	For tap water, any residual chlorine levels must be reduced before use. Recommended ranges: ammonia (NH_3_/NH_4_**^+^**) < 0.1 mg/l, nitrite (NO_2_**^–^**) < 0.3 mg/l and nitrate (NO_3_**^–^**) < 25 mg/l; pH 6.5–8; conductivity between 150 and 1700 µS/cm and general water hardness (dGH) between 3–8. For experimental situations and for limited periods other values can be tolerated as long as they are not fluctuating. Monitoring and documentation of pH and conductivity is typically done on a daily basis and of nitrogen compounds on a weekly basis.	12, 24, 25, 29
Stocking density	For long-term housing a standard of 4–10 adult fish/l will help to maintain low stress levels and good water quality. For embryos, a recommended upper limit is 100/35 ml in a 9 cm Petri dish, and for 5–10 dpf larvae, up to 250/l. Higher and lower densities, including keeping single fish, can be tolerated for limited periods.	29, 37, 38, 39, 40
Environmental enrichment	Zebrafish are a shoaling species and should be kept in groups. Single zebrafish housing should be limited to restricted periods. At present, there is a lack of evidence for improvements of fish welfare to support the recommendation of routine physical enrichment.	41, 42, 43, 44, 45, 46
Feeding	Feed zebrafish two to three times a day with a combination of dry and live feeds. However, the omission of live feeds and lower or higher feeding frequencies are shown to be fully acceptable for animal welfare.	24, 25, 47, 48
Breeding, identification, nomenclature	Avoid repeated inbreeding from sibling matings. As an inventory measure, date of fertilisation, number of fish and key genetic information (transgenesis, mutation) needs to be traceable. Follow recommendations by the zebrafish nomenclature committee (see *zfin.org*).	24, 25, 49, 50
Sanitation of equipment and hygiene	Standard operating procedures need to be in place to ensure that possible pathogens cannot spread from one tank to the next. Disinfect equipment after each use. Use of hand disinfection routines to avoid cross contamination and zoonotic infections is recommended.	

### Transportation and reception

Exchange of fish between laboratories usually poses three main challenges: organising safe shipment of fish, ensuring compliance to fish welfare as well as to national and international legislations, and finally avoiding spreading pathogens between laboratories. For genetically modified zebrafish lines, regulations related to a transgenic or genetically modified organism’s (GMO) biosafety and restrictions must also be fulfilled. If available, documentation of health monitoring and relevant husbandry parameters used for the originator population should precede the shipment.^6,22^

During shipment, the challenge is to keep temperature and water quality parameters within a suitable range in particular with respect to oxygen, CO_2_ and nitrogen.^25^ Shipment times should be as short as possible, and it is generally easier to send and receive embryos than adult fish.

Embryos prepared for shipping should be surface-disinfected using sodium hypochlorite (bleaching) or iodine solutions prior to shipment.^14^ Povidone-iodine has been proven effective against *Mycobacterium* species.^28^ At reception, embryos/larvae should be visually inspected and live embryos should be transferred to a small tank or Petri dish.

Adult fish shipment requires a relatively low density of fish (two adult fish/0.5 l) and a 1:1 or higher ratio of air or oxygen to water volume in each container.^25^ Feeding should be withheld from juveniles and adults for 24 hours prior to packaging in order to reduce excretion and avoid water fouling in the container. For shipment longer than a day, it is recommended to add ammonia binder to the shipping water, to further limit health risks to the fish.^12^

Reception of adult fish must be well organised. During transport the water quality will have decreased. Upon arrival, fish should first be visually examined and if any animals look unhealthy, it is wise to consider not introducing them into the facility, not even into a quarantine system. The temperature of the transport water should be adjusted by floating the arrival container in the destination tank without opening the container. The CO_2_ exhaled by fish during transport is partly dissolved in water, acidifying the water and reducing the toxicity of the ammonia (NH_3_/NH_4_^+^) accumulated during transport; a sudden release of CO_2_ occurring at the opening of the container induces an ammonia toxicity increase. In order to avoid ammonia poisoning, it is vital that adult fish, after temperature adaptation, are removed with a net from the transport bag immediately after opening and transferred into the reception tank without transferring the water from the transport bag. New fish should be monitored closely for at least two weeks.

### Quarantine for biosecure introduction of new strains/fish

After safe reception of embryos or fish, the next challenge is to avoid the spread of potential pathogens into the main section of a facility. While egg surface disinfection (bleaching)^14^ strongly improves biosecurity, it does not suffice to safeguard against dissemination of intracellular pathogens.^28^ It is strongly recommended to keep and breed newly imported animals under quarantine containment to limit the risk of pathogen dissemination into the main facility. For laboratories with only a few tanks, it is appropriate to dedicate one tank with its own water circulation as a quarantine unit, to reduce the risk of spreading pathogens to the established fish when introducing new lines. The quarantine unit should be kept as separated as possible from other units, preferably in a different room. Only dedicated equipment should be used in the quarantine unit, be clearly identifiable and never be mixed with the equipment of the main facility. We recommend both close observation of the health status of individual fish held in the quarantine system and exclusively using bleached offspring from quarantined healthy-appearing parents for transfer into the main facility.

### Water and housing systems

Decades of experience have proven that maintaining zebrafish is a relatively straightforward task.^25^ In most cases, commercially available tank systems are used, which come fully integrated with filter systems, germicidal irradiation (UVC) and light and temperature control units.^29^ These systems contain either permanently installed tanks (glass or polycarbonate), or units that can be removed from the main water supply and reconnected again depending on specific needs. Standard fish tanks typically vary between 1 and 10 l. Most systems rely on a recirculating water system in which pumps feed water into the tanks and, through an overflow system, remove an equal amount of water. The waste water is partially purified before being recirculated.

Flow-through systems depend on consistently high-quality source water all year round and an outflow capacity of correspondingly large volumes. Energy consumption is higher than for recirculating systems due to the need to constantly heat, condition and mix large amounts of water. From a recent survey, almost 20% of laboratories reported using flow-through systems.^19^ Flow-through set-ups might have an advantage in terms of disease control.

### Temperature

Fish are poikilothermic and the zebrafish is a tropical species (although many natural habitats are temperate, Figure 1).^7,8^ The commonly used references to developmental stages relate to hpf determined by the speed of development at 28.5℃.^30^ Temperature influences water chemistry and animal physiology.^29^ The solubility of oxygen in water decreases as the temperature rises. However, recirculating systems do not require special measures to introduce oxygen, as the constant water motion results in sufficient oxygenation. Due to several practical considerations and aims towards standardised husbandry procedures, the temperature of housing water in different facilities is typically kept in a range between 24–29℃. The importance of avoiding sudden changes in temperature must be emphasised. The tolerance of zebrafish for both lower and higher temperatures is well documented^31^ and a wider range of temperatures is not in conflict with animal welfare standards. At lower temperatures, embryos/larvae grow more slowly but for adult fish there are no known behavioural differences at the temperatures in the proposed range. Therefore, during certain experimental conditions such as, for example, the development of vaccines against virus infections in cold-water species,^32^ lower temperatures (15℃) may be applied. During studies of temperature effects on sex determination, higher temperatures (36℃) have been applied.^33^ Since the experimental temperature regime may affect early development and physiological processes, the temperature used for growth and maintenance should be stated in the Materials and Methods section of publications.

### Dark–light cycle

In nature, light conditions vary with seasons and weather. Modern laboratory facilities present the fish with a static dark–light (D–L) cycle (commonly 10 hours dark, 14 hours light, optionally with gradual decrease and increase in light intensity mimicking dusk and sunrise). Using other settings (such as 12:12 D–L cycle) will not affect animal welfare as such, but may influence physiological processes, for example spawning frequency (and hence breeding success). Light cycle parameters should be stated in the Materials and Methods section of publications. The intensity of light should be as uniform as possible across tanks and intensities should be adjusted to between 54 and 334 lux at the front of the tank.^34^ A recent report demonstrates the importance of a complete darkness period for reproductive performance.^35^ Furthermore, several studies suggest that a D–L regimen is important also for early embryos^36^, hence an (albeit possibly relaxed) D–L cycle is advisable.

### Water quality

Depending on the local water supply used for recirculating water systems, some laboratories can use tap water without major amendments. Chlorine still needs to be removed, as levels safe for humans (*c.* 0.1 mg/l) are toxic for fish. Constant stirring and aerating of the water for 24 hours prior to use is sufficient to remove chlorine, whereas water sanitised with chloramine (which is less volatile) needs to be filtered through active charcoal. In most facilities, the water chemistry needs to be adjusted before use. Frequently, tap water hardness is too high and needs to be adjusted by mixing with deionised water, or too low, requiring addition of salts. Many facilities only use conditioned deionised water. Reverse osmosis (RO) is commonly used for deionisation and sea salt, calcium chloride and sodium bicarbonate are then added to achieve the desired conductivity, hardness and pH.^25^ Water softening using ion exchange resin replaces divalent ions (such as calcium and magnesium) with sodium, and so delivering a soft (no calcium) but salty water. Hence, softened water needs to be treated by RO to remove sodium.

Water hardness (general hardness (GH) describes the concentration of divalent metal ions such as Ca^2+^ and Mg^2+^; carbonate hardness (KH) describes the concentration of carbonates such CaCO_3_ and MgCO_3_) and conductivity are interdependent water parameters. Depending on the way the system water has been produced, the conductivity is mainly determined by the quantity of sodium and chloride (sea salt reconstituted water) or calcium and carbonate (‘cichlid salt’ reconstituted water and tap water/RO water mix). Conductivity is kept at different levels in different facilities, typically above 150 and up to 1700 µS/cm.^12,24^ Degrees (d) of water hardness, dGH and dKH, is kept in a range between 3 and 8 and commonly around 4–5. Concerning pH, a suitable range is between pH 6.5 to 8 (in natural habitats pH varies between 6 to 10; Figure 1). For closed systems with recirculating water, the biofilter will work better at pH levels above 7. The outgoing water in recirculating systems is purified through a combination of mechanical and biological filters and then exposed to UVC irradiation, before it is reintroduced into tanks. The biological filter consists of nitrifying bacteria cultivated on a large surface (commonly provided by plastic beads) and is responsible for the oxidation of ammonia (NH_3_/NH_4_^+^) into nitrite (NO_2_^–^) and further into less toxic nitrate (NO_3_^–^). A well-dimensioned biofilter keeps levels of total ammonia < 0.1 mg/l, nitrite < 0.3 mg/l and nitrate < 25 mg/l. A proportion, normally 5–10% (occasionally up to 20%) of the water in each system should be exchanged with new water on a daily basis, in order to keep nitrate and other harmful pollutant levels low. Monitoring and documentation of pH and conductivity should typically be done on a daily basis, and of nitrogen compounds on a weekly basis.

### Stocking density

Embryos hatch around 60 hpf and settle over the bottom of the Petri dish/tank until roughly 5 days post fertilisation (dpf) when the swim bladder has developed sufficiently to allow swimming.^17^ Body mass, rather than numbers of animals, sets the limits for fish/l density recommendations. Thus, the density of juveniles can be higher than the corresponding figures given for adults. One aspect of importance is that lower densities support sex differentiation towards females and higher densities the development of males.^37^ This long-established phenomenon, supported by empirical data from many laboratories, has recently been challenged by a study claiming that sexual determination is more complex, being controlled primarily by genetics rather than by environmental factors.^38^

In modern fish facilities, equipped with efficient and standardised water quality measures, fish are often maintained at densities of 4–10 adult fish/l. There is evidence in the literature that fish densities between 3 and 12 adult fish/l in tanks without environmental enrichment (see below) show no impact on reproductive performance.^29,39,40^ Since other factors (feed quality, water parameters, etc.) also play a role and since these parameters will vary between facilities, maintaining water quality is key for standard stocking densities. Higher numbers might not necessarily cause a problem in terms of physiological parameters for the fish but make visual inspection of fish more demanding. At times, it is necessary to identify a single fish (for example to identify transgenic or mutant carriers). Currently, the best approach is to isolate them from their siblings, as marking of individual zebrafish has proven impractical to date. However, isolation for an extended period of time is thought to be detrimental to zebrafish welfare, hence isolation time needs to be restricted to a minimum.

### Environmental enrichment

In general, observing normal behaviour patterns and the absence of any signs of illness or stress among the fish suggests that well-being is maintained. Two commonly used laboratory measures for animal welfare are reproductive success and plasma (or water) cortisol levels. Changes in the D–L cycle, water conditions (e.g. rise in ammonia levels) or presence of parasites (e.g. nematodes) are associated with reduced egg production. With regard to the stress hormone cortisol, it has been shown that crowded and non-fed zebrafish release higher levels of this hormone.^41^ Cortisol level can be assessed as total body content or by analysing the tank water the fish are maintained in.^40^ However, a recent review of cortisol in finfish suggests that single measurements of cortisol levels in blood or water are unable to differentiate between adaptive and maladaptive responses, and might therefore be poor indicators of fish welfare.^42^

Physical enrichment has become mandatory for rodents, but the benefits for zebrafish are still disputed for several reasons. There is no experimental evidence suggesting any clear improvement of animal well-being by the addition of physical enrichment in zebrafish tanks.^43^ Plastic plants seem to have little effect on group-housed zebrafish, but might be beneficial for single-housed animals.^44^ However using plastic plants for enrichment raises the issue of risk of toxicological effects from additives in plastic materials. Tests would have to be performed to ensure that there are no plastic microparticles or harmful substances such as bisphenol A/B accumulating in either the fish or in critical parts of the water system (e.g. magnetic valves), endangering fish health and safe execution of technical operations. Indeed, adult fish have been observed to nibble on plastic grass and to ingest it (B. Schmid, personal communication 2018). Another consequence is hygienic disadvantages caused by increased risk of biofilm development and the improved environmental conditions for microbial overgrowth it generates as plastic accessories usually cannot be autoclaved or bleached. Lastly, zebrafish tend to spawn as soon as there are any uneven surfaces or objects in the environment and the long-term effect of, in extreme cases, daily spawning needs to be assessed before the pros and cons of tank enrichment can be evaluated. Providing live feeds like *Artemia* stimulate natural prey-capture behaviour and is considered to constitute an important environmental enrichment. One study reports that fish exhibited a preference for gravel substrate or images of gravel on the bottom of the tank, but stress appeared unaltered compared to fish in barren glass tanks.^43,45^ The latter type of ‘visual’ enrichment would have the significant advantage of not obstructing the work to keep fish tanks clean, something that is invariably an issue with physical enrichment.

Even though activities in the area of tank enrichment have increased, at present there is not sufficient information to formulate a recommendation concerning this issue.

In the wild, zebrafish do not appear to avoid open water areas, and there is no clear evidence that fish are stressed in tanks without constant enrichment. It is interesting to note that shoaling behaviour can be observed among fish that are separated by glass,^46^ remaining in visual but not in physical contact, which suggests neighbouring tanks as being potential stress and aggression reducers.

### Feeding

It is generally accepted that a combination of live feeds and processed dry feeds improves growth, generation time and reproductive performance, all positive indications of well-being. Dry feed diets are generally assumed to be nutritionally complete, whereas live feed and the associated fish prey-capture behaviour have an enrichment effect.^24,25,47^ Formulated diets simplify feed delivery, storage and preparation. There are several organisms used as live feed, for example *Paramecium caudatum*, *Tetrahymena*, rotifers (*Brachionus* sp.), and *Artemia nauplii*. The gape size of 120 hpf larvae allows them to consume smaller diameter *Paramecium* and rotifers, but usually not *Artemia*. Using saltwater rotifers (*Brachionus plicatilis*), including a static rotifer–larvae ‘polyculture’ method has been reported effective for feeding of 5–9 dpf larvae.^24,48^ Naturally, since live organisms can be vectors for pathogens, care must be taken to ensure pathogen-free sources.

The frequency of feeding should also be adjusted according to the developmental stage of fish. Larvae grow quickly and should be fed, if possible, two or three times per day with live feed. Feeding can be carried out manually or automatically. Automatic feeding robots can dispense both dry and live feeds and provide a well-defined amount of feed; however, having differing numbers of animals in tanks requires proper programming. If using automated feeding, the importance of daily visual inspections of fish tanks by staff must be emphasised.

The rationale behind a frequent feeding regimen is based on the fact that the zebrafish lacks a stomach and feeding 2–3 times per day is common practice, one of which should include live feed for enrichment. However, from one month onwards feeding fish with dry feed only once per day is reported to have no negative effects on welfare indicators like growth and, for mature fish, reproduction.^47^

### Breeding, identification, nomenclature

Among the attributes that make the zebrafish a great model species is its high fecundity. A female can spawn hundreds of eggs per single mating. It is however recommended to set up fish for spawning with a recovery period of at least one week to allow for sufficient regeneration and maturation of new ova. Adults periodically not used for embryo production should be kept as mixed sex groups to allow natural breeding behaviour and avoid egg-associated inflammation of the oviduct (‘egg bound’ females).

Genetic management of fish colonies is important to minimise deleterious effects of inbreeding and to reduce the loss of genetic diversity within a population over time. For space and cost reasons, distinct strains and lines of fish are often kept in relatively small groups. To avoid inbreeding depression, each new generation should be produced by an outcross, and sibling matings should be performed only when absolutely necessary.^24,25,49^ Inbreeding signs include loss of fecundity, premature ageing of adults and difficulties in obtaining good survival rates when raising fish.

Rearing of embryos and larvae represents a key issue in a zebrafish facility.^50^ Embryos are kept in embryo medium (often containing 0.5 mg/l methylene blue, to reduce fungal infections), at a stocking density of up to 100 embryos/ 35 ml in a 9 cm diameter Petri dish, at 28.5 ± 0.5℃ in D–L cycle. Use of autoclaved medium or otherwise sterilised water is necessary. Any remains present with the eggs after spawning should be carefully removed (faeces, scales, dead and unfertilised eggs) prior to transfer into the dish. The medium should be changed regularly, and non-viable embryos, dead eggs or chorion remains should be removed at the same time.

At around 120 hpf the digestive tract has developed, the anus has opened, the swim bladder is inflated, and larvae are able to swim and feed independently.^17^ Early larvae are often maintained in small tanks with the water flow turned off at up to 250 larvae/l. When reaching an appropriate size, the juveniles are transferred to aquaria with water exchange. The period to reach sexual maturity varies between 2 to 4 months depending on the environment, feeding and husbandry conditions.

All adult fish in a facility need to be tracked via the use of an appropriate inventory database system that allows central monitoring of animal numbers, breeding history, characteristics of a given strain and other vital information. Minimum parameters that should be tracked are name of fish strain/line, the date of fertilisation (DoF), number of fish (initially entered into the system/current number calculated by subtraction of dead fish) and molecular modifications/genetic information. Recommendations by the Zebrafish Nomenclature Committee (see *zfin.org*) should be followed, but well defined, abbreviated working names constitute a practical day-to-day solution.

### Sanitisation of equipment and hygiene

A clean environment is essential for maintaining a high standard of animal health and welfare. To achieve this goal, special care needs to be taken to avoid cross contamination during routine husbandry procedures, since many diseases can be spread through physical contact between individual fish, tanks and water systems. Any piece of equipment in physical contact with fish (such as nets, mating boxes, etc.) should be dedicated to one specific system and sanitised periodically. Chemical sanitisation is possible, but care needs to be taken (e.g. sufficient rinsing with water) to avoid contaminating the water with chemicals. An alternative simple and safe cleaning method is heating at a minimum of 60℃ for at least one hour (shorter times when using higher temperatures), by the use of dishwashers, jam cookers, etc. Autoclaving is more demanding to achieve and often not practical but provides sterilisation when needed. Equipment used for quarantine units needs to be isolated from equipment used at the main facility. As a general rule, staff, material and work movement should be carefully considered so as to reduce contamination. Cleaner areas/tasks should be attended to before dirtier ones. For example, feeding and cleaning of the main facility will be completed prior to attending to the quarantine facility.

Growth of algae needs to be monitored and must not interfere with the visual inspection of fish. It is also an indicator of biofilm, which can harbour harmful pathogens and should be periodically removed. Use of gloves and/or appropriate hand disinfection routines are important in order to avoid cross contamination between fish populations and exposure of facility staff and researchers to zoonotic infections.

## Discussion

The purpose of these guidelines is to set a standard for zebrafish husbandry and to provide a basis for facility heads, husbandry personnel, veterinarians and other stakeholders to discuss further improvements in zebrafish husbandry. The current EU Directive 2010/63 does not stipulate recommended procedures for small teleost species such as zebrafish. Hence, the present document provided by this FELASA working group intends to fill this void.

In a laboratory setting the aim is to maintain zebrafish in a 24/7 controlled environment. Parameters for well-being are reproductive success, growth and the absence of signs of illness or excessive stress. The knowledge about the plasticity that zebrafish display in nature, tells us that the current variations in fish facility conditions do not challenge animal welfare as such, and also that those ranges of husbandry parameters (e.g. for pH and temperature) mimic natural situations.

A central theme in the 2010/63/EU Directive is euthanasia. This important part of husbandry routines, dealt with at a recent EUFishBioMed workshop,^27^ together with issues related to background pathology and health monitoring could not be dealt with in this report due to space constraints, but will be discussed elsewhere.

Further, there is need for more research on physiologically normal cortisol response patterns (amplitude, time course) versus abnormal patterns for objectively indicating impaired well-being. At present, there is no good option to measure well-being of an individual fish or a population in a quantifiable manner. In order to avoid the housing of single fish causing stress, and to enable cohabitant housing with identical conditions of study groups, there is an unmet need for novel marking methods.

The range of conditions that has been shown to be physiologically acceptable for zebrafish, both in the laboratory and in their natural habitat,^8^ are rather broader than those commonly used for general housing (Figure 1). Thus, deviations from the recommended ranges should not *per se* be considered violations of animal welfare as long as there are either no indications for compromised well-being, or the conditions are scientifically necessary and ethically justifiable.

We trust that these recommendations for guidelines will help ensure good welfare and improve reproducibility of zebrafish research in Europe and worldwide.
